# Focusing with saw-tooth refractive lenses at a high-energy X-ray beamline

**DOI:** 10.1107/S1600577520003665

**Published:** 2020-04-07

**Authors:** S. D. Shastri, P. Kenesei, A. Mashayekhi, P. A Shade

**Affiliations:** a Advanced Photon Source, Argonne National Laboratory, Lemont, IL, USA; b Materials and Manufacturing Directorate, AFRL, Wright-Patterson AFB, OH, USA

**Keywords:** X-ray refractive lenses, saw-tooth lenses, X-ray optics, X-ray focusing, high-energy X-rays

## Abstract

The Advanced Photon Source 1-ID beamline, operating in the 40–140 keV X-ray energy range, has successfully employed continuously tunable saw-tooth refractive lenses to routinely deliver beams focused in both one and two dimensions to experiments for over 15 years. The practical experience of implementing such lenses, made of silicon and aluminium, is presented, including their properties, control, alignment, and diagnostic methods, achieving ∼1 µm focusing (vertically). Ongoing development and prospects towards submicrometre focusing at these high energies are also mentioned.

## Abstract 2

The implementation of saw-tooth refractive lenses at the APS 1-ID high-energy X-ray beamline is presented.

## 1. Introduction   

The 1-ID X-ray beamline exploits the high energy (7 GeV) of the Advanced Photon Source (APS) storage ring’s electron beam, its low emittance, a short-period undulator source and optics optimized for high-energy X-rays, to provide high-brilliance beams in the 40–140 keV photon energy range for scattering studies of materials. The interaction of such X-rays with matter is characterized by low attenuation, small scattering angles, and large reciprocal space access, making them well suited as a bulk probe and for geometrically constraining or extreme sample environments. A significant portion of the beamline’s scope pertains to studying the microstructure and evolution of engineering materials with high spatial resolution, *e.g.* obtaining three-dimensional grain maps of polycrystalline materials, giving position, shape, crystallographic orientation and strain state, and often tracking these parameters for thousands of grains while undergoing micromechanical changes under applied stimuli. High-spatial-resolution studies are often conducted through a combination of complementary techniques using both focused and unfocused beams on the same specimen. Focused beam techniques would include near-field high-energy diffraction microscopy (nf-HEDM; Suter *et al.*, 2006 ▸), diffraction tomography (Birkbak *et al.*, 2017 ▸) and coherent diffraction imaging (CDI). Unfocused beams are used in conventional tomography and far-field high-energy diffraction microscopy (ff-HEDM; Lienert *et al.*, 2011 ▸). Enabling such a suite of techniques makes in-line focusing optics desirable, resulting in an invariant beam position for the line(1D)-focused, point(2D)-focused and unfocused configurations. For mainly this reason, Kirkpatrick–Baez reflection optics are not employed, although they are achromatic and thereby easily accommodate energy-tunability (if based on total external reflection, and not multilayers). Also, small focal spot positions are susceptible to the angular stability of reflective optics, unlike in-line optics. Fresnel-zone-based optics (*e.g.* zone plates and multilayer Laue lenses) operate in-line, but have other diffraction-order halos, whose elimination requires order-sorting apertures, which are difficult to fabricate for high X-ray energies. Furthernore, they are not tunable, a characteristic defined as the capability of preserving a fixed working (focal) distance while changing energy, as would be desirable for a beamline like 1-ID that offers continuous energy tunability over a wide range. Kinoforms, which can be viewed as properly blazed zone plates to eliminate the unwanted orders, have successfully demonstrated submicrometre focusing at high energies (Shastri *et al.*, 2014 ▸). However, the of lack of tunability characteristic remains.

Achieving tunability in an in-line configuration could lead one to consider compound refractive lenses (CRLs), which achieve focusing by the cumulative refractive action of concave lens elements in a linear array. By varying the number of elements, tunability is attained in a discrete way, which may be viewed as effectively continuous only if the resulting increments in focal distance are within the depth of focus. For high energies, given that the number of elements increases quadratically with energy, actuator-based mechanisms that facilitate varying their number (Vaughan *et al.*, 2011 ▸; Duller *et al.*, 2016 ▸; Shu *et al.*, 2018 ▸) can be bulky, especially if both 1D and 2D focusing capabilities are desired, as they are implemented by different types of elements. Etched 1D focusing CRLs on Si wafers (Snigirev *et al.*, 2009 ▸) can be compact. However, their small (submillimetre) etch depths (in the direction perpendicular to focusing) preclude providing long (few millimetres) line foci, as needed for certain techniques, such as nf-HEDM.

This article reports on the practical experience with a specific type of refractive lens, namely saw-tooth refractive lenses (SRLs), at APS 1-ID, where such lenses have been in routine operation for 1D and 2D focusing of high-energy X-rays for over 15 years. These lenses are in-line, continuously tunable in energy (or focal length), effectively parabolic (*i.e.* having that desired profile in thickness projected along the beam), and have zero attenuation on-axis (unlike CRLs’ wall-elements). In 1D focusing applications, very large spatial acceptances of many millimetres are achievable (in contrast to etched Si CRLs), providing long line foci when needed. The implementation of SRLs is discussed with regard to their features, control, alignment and diagnostics, with focusing results down to ∼1 µm levels. Development towards submicrometre focusing is mentioned here, but details of those efforts will be presented elsewhere.

## 2. Saw-tooth lens operating principles   

**Figure 1:**
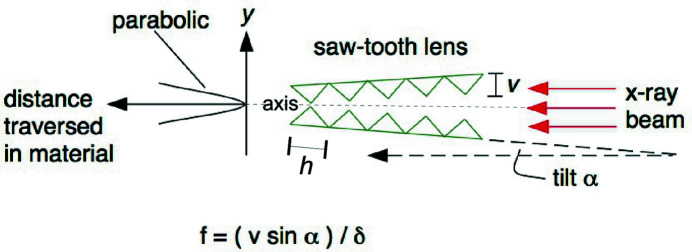
A pair of triangular saw-tooth structures tapered symmetrically ± α about the beam axis presents a parabolic transmission thickness profile.

SRLs operate on the principle that a linear, triangular saw-tooth structure, in an overall grazing-incidence setting with respect to a beam, presents a parabolic thickness profile (Cederström *et al.*, 2000 ▸, 2002 ▸). A full symmetric parabolic profile requires placement of two such saw-tooth structures face-to-face, canted symmetrically about the optical axis (Fig. 1 ▸). Individual teeth can be viewed as refractive prisms imparting equal angular deflections to the X-rays. A ray receives a number of prism deflections that is proportional to its distance *y* off-axis, resulting in all rays being directed to a single focus, whose focal distance away depends on the single-prism deflection, saw-tooth period, and the grazing angle of the array.

**Figure 2:**
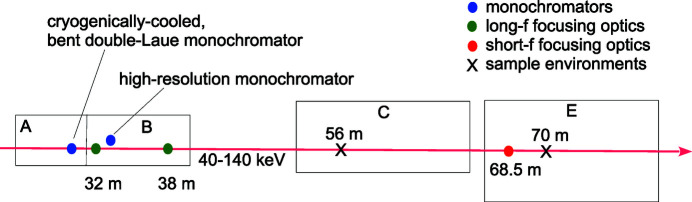
Plan view layout of the APS 1-ID high-energy X-ray beamline with distances from the undulator source indicated.

Alternatively, the parabolic profile feature is revealed if one notes that, for an off-axis ray with *y* that just barely misses the tip of the (*m* + 1)th tooth from the axis, it will go through a thickness Δ*z* of tooth *m*, 2Δ*z* of tooth *m* − 1, 3Δ*z* of tooth *m* − 2,… and *m*Δ*z* of tooth 1, resulting in a total traversed thickness 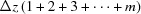, which is an arithmetic series that grows quadratically with *m*, and hence also with *y*. Depending on the period *h* and the taper angle α with respect to the beam, the parabola is approximated in a very fine, piecewise-linear but continuous fashion (with discontinuities in the derivative), assuming the teeth narrow down to ideal edges. The fineness of this sampling interval 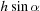, corresponding to the elevation difference between adjacent teeth, as viewed along the grazing-incidence beam, becomes a refractive discreteness aberration contribution to the geometrical optics point-spread function of the lens. In addition to no thickness attenuation on-axis, the focal length of an SRL is easily tuned by symmetric adjustment of the two pieces’ taper angles, which alters the vertex curvature-radius *R* of the effective parabola, through the relation *R* = 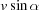, where *v* is the tooth height. The focal length is then given by the expression for the single plano-concave refractive lens, *f* = *R*/δ, where δ = 1 − *n* quantifies the decrement of the material’s refractive index from that of vacuum, hence giving *f* = 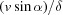. One should note that the two-piece SRL arrangement depicted in Fig. 1 ▸ accomplishes focusing in one direction only, *i.e.* in the plane of the figure. 2D focusing, as will be shown later, requires adding a second, similar lens set-up oriented perpendicularly to focus in the other direction. The maximum spatial acceptance of one lens piece, beyond which the parabolic profile ceases, is given by the tooth height *v*, provided the piece is long enough to accommodate the beam footprint 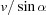 at the operating grazing incidence; otherwise it is limited to the *length* 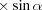. For the two-piece SRL, the acceptance is doubled, the maximum possible being 2*v*. Considering the effect of exponential attenuation by a parabolic profile, the Gaussian physical aperture of SRLs is equivalent to that obtained by stacking many CRL elements (even if a single element has large acceptance), except that the latter imposes nonzero attenuation on-axis.

The Si and Al SRLs discussed here have saw-tooth parameters in the ranges of 6–16 cm lengths, 100–200 µm tooth heights, 100–500 µm periods and large 6 mm lateral widths. For such parameters and 50–100 keV X-rays, the piecewise-linear approximation to the ideal parabola occurs in micrometre- to 100 nm-step segments of *y*, depending on the value of α, which lies between a few hundredths to a few tenths of a degree, for focal lengths *f* in the 1–20 m range. SRLs are well suited to high-energy X-rays (>50 keV) (Shastri *et al.*, 2007 ▸), which reduce α to assume values that make the refractive discreteness aberration 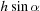 appropriately small compared with the spot size (*e.g.* a few 100 nanometres relative to a few micrometres focus). Significantly lowering the energy strongly increases 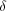 (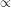 wavelength^2^), in turn increasing α ≃ *f*δ/*v*, and thereby coarsening the approximation to the parabola and increasing the refractive discreteness aberration 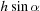. This can be partially mitigated by making SRLs for low energies out of light elements, such as Be (Ribbing *et al.*, 2003 ▸) or Li (Dufresne *et al.*, 2001 ▸). Medium-*Z* materials like Si and Al are practical and well suited for the high photon energies of interest here. Due to the dominance of Compton over photoelectric attenuation in this wavelength range, using lower-*Z* materials for refractive lenses yields at best slight transmission-aperture improvements, at the expense of inconveniences and sensitivities of longer devices required by the weaker refraction per unit length associated with lower density. Fabrication issues aside, diamond, in principle, is considered ideal for refractive optics due to its high density and low attenuation. However, for SRLs, diamond requires careful consideration, as its high refractive strength δ would result in greater inclination angle α and refractive discreteness aberration. So diamond SRLs would be suitable only at very high energy (>100 keV) or sufficiently short focal length (*f* < 0.3 m) conditions, where appropriately low operating angles α are restored.

## 3. APS 1-ID beamline – configuration and source   

This section describes aspects of the APS 1-ID beamline layout, source, and optics relevant to the focusing discussions here. 1-ID is a single-branch, in-line beamline operating in the 40–140 keV range (Fig. 2 ▸) using a cryogenically cooled, fixed-vertical-offset, bent double-Laue monochromator in the A station at 29.5 m from the source, providing ∼10^−3^ bandwidth (Shastri *et al.*, 2002 ▸). It comprises two vertically diffracting, asymmetric Laue crystals bent to sequential Rowland (*i.e.* inverse-Cauchois) conditions with respect to the source. SRL long-focal-length systems, located at 32 m and 38 m in the B station, are capable of delivering focused beams to the sample positions in the C and E end-stations at 56 m and 70 m. Experiments in the E station also utilize short-focal-length focusing from the SRL set-up at ∼68.5 m within E.

The upstream focusing optics at 32 m are sometimes also employed in a vertically collimating mode to enhance the throughput of an optional, subsequent, narrow-angular-acceptance, high-energy-resolution, four-reflection monochromator (Shastri, 2004 ▸) that further reduces the bandwidth to 10^−4^–10^−5^ levels. This enables applications like resonant pair-distribution-function measurements at heavy-element *K* edges, very-far-field HEDM, and Bragg CDI. The last two methods entail focusing the beam from the high-resolution monochromator into the E station, using the SRL set-up at 38 m or 68.5 m, achieving small beams combined with the high-reciprocal-space resolution from the reduced energy spread to examine sub-grain features such as dislocations and intra-granular strains.

The beamline’s undulator radiation source has evolved over time from various permanent-magnet devices to the present 1.1 m-long, 1.8 cm-period superconducting device (Ivanyushenkov *et al.*, 2017 ▸). The FWHM vertical and horizontal sizes of the electron source are typically 26 µm and 635 µm, respectively.

**Figure 3:**
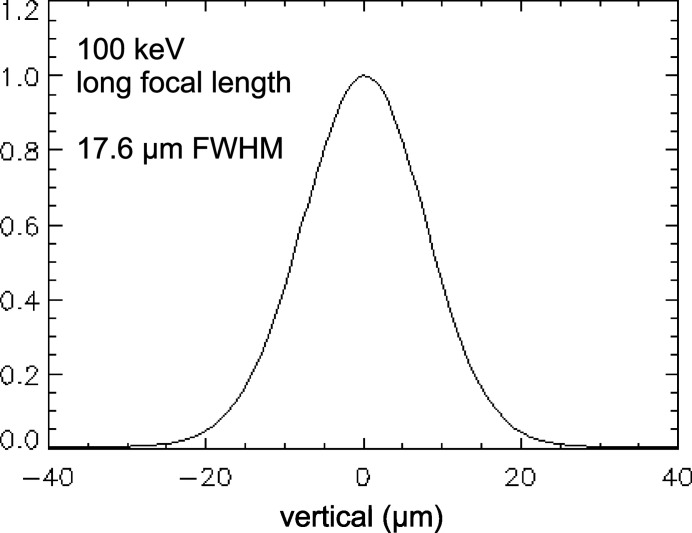
Vertical focus profile for a long-focal-length configuration, with 100 keV X-rays focused by Si lenses from the 32 m location (B station) to the 56 m location (C station), as illustrated in the Fig. 2 ▸ layout.

The performance of focusing optics depends not only on its intrinsic quality but also on the extent to which the beamline’s components preserve the source size. Despite the monochromator’s diffraction by asymmetric Laue crystals and their bending in the vertical direction, the source properties and ray propagation in the vertical plane are well preserved, as has been routinely confirmed by achieving close to expected focal spot sizes (<30% discrepancy) at long focal distances using various focusing optics. This is due to an effect in which the second bent crystal compensates the perturbation of the X-ray phase space distribution imparted by the first bent crystal’s Borrmann fan (Lienert *et al.*, 2001 ▸). Mechanical vibrations are also of concern. Testing for source size preservation in propagation through beamline components can be conducted at long focal lengths, where the quality requirements of focusing optics are more relaxed to meet. At 1-ID, source size preservation is shown in Fig. 3 ▸, which shows the focus profile from a vertically focusing Si SRL system (upright and inverted pair) at the 32 m position (in B) focusing 100 keV X-rays to the 56 m position (in C). The Si lenses were 6 cm long, with isosceles teeth of 200 µm height and 283 µm period. The measured vertical size of 18.3 µm FWHM, when corrected (deconvolved) for the 5 µm wide scanning slit, implies a spot size of (18.3^2^ − 5^2^)^1/2^ µm = 17.6 µm, in agreement with the degmagnification of the source [(56 − 32)/32] 2.35σ_*y*_ = 17.6 µm, where the RMS vertical source size was σ_*y*_ = 10 µm at that time. Although the agreement here is exact, long-focal-length spot sizes can be anywhere up to 30% larger, the discrepancies being attributed to the possibility of the eccentric elliptical source being spatially rotated by angles up to χ = 2° (Dufresne & Khounsary, 2007 ▸). Such tilts have the consequence of an amount 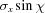 from the large horizontal source size σ_*x*_ contributing to the vertical source size. For 1D focusing, the transverse inclination of the lens defines the orientation of the line focus. A rotated source combined with a transversely untilted lens produces a line focus that is un­rotated, but broadened. So the effective RMS vertical source size becomes 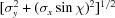, which amounts to 1.3σ_*y*_ for a tilt of χ = 2° and the APS source’s aspect ratio σ_*x*_/σ_*y*_ ≃ 25.

## 4. Short focal length – Si lenses   

**Figure 4:**
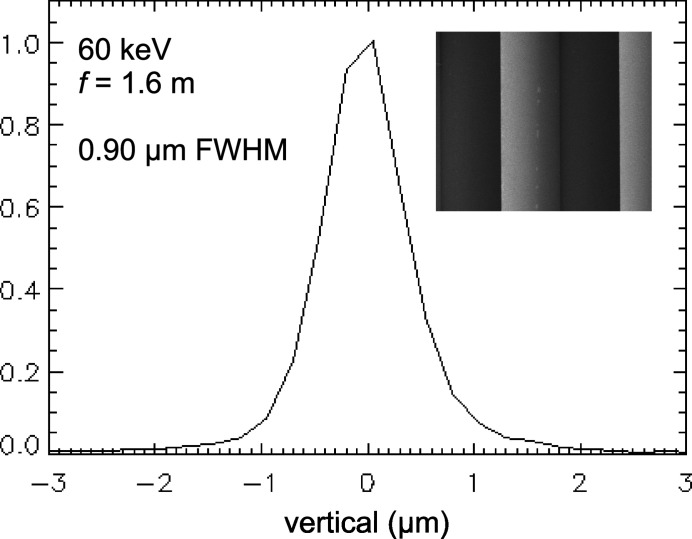
Vertical focus profile for a short-focal-length configuration, with 60 keV X-rays focused by Si lenses at 68–70 m in the E station. The inset is an SEM image of a device looking down on teeth having 100 µm height and 142 µm period.

Single-crystal Si SRLs have been fabricated through two processes – anisotropic etching (Ribbing *et al.*, 2003 ▸) and dicing (Said & Shastri, 2010 ▸). The Si SRLs discussed in this article were produced by anisotropic etching, through the efforts of the former reference’s authors. Fig. 4 ▸ shows the focus profile from Si SRLs (upright and inverted pair) placed at 68.4 m in the E station, focusing vertically to the 70 m position (see Fig. 2 ▸), corresponding to *f* ≃ 1.6 m. A 60 keV X-ray beam of size 300 µm × 200 µm [horizontal (H) × vertical (V)] was incident at grazing angle α = 0.12° on the lenses, which were 9 cm long, with isosceles teeth of 100 µm height and 142 µm period. The measured focus was 0.90 µm FWHM, determined by detecting Au *L*-fluorescence from scanning a fine, 245 nm tall Au wire through the focus (described in more detail in a subsequent section). By comparing the fluorescence signals with and without focusing, a flux density gain of 140 was determined (70 from each piece), consistent with the 124 µm effective vertical transmission aperture calculated for the SRL pair.

The expected vertical focus size is 0.74 µm, with contributions of 0.60 µm, 0.17 µm, 0.10 µm, 0.30 µm and 0.245 µm from source demagnification, diffraction limit, chromatic aberration, refractive discreteness aberration and the fluorescence profiler, respectively. Under these same conditions, focal widths up to 1.3 µm are observed. So, in addition to lens imperfections, the previously discussed electron source tilt, which can vary from time to time, also contributes to wider line foci. Line-focused beams, generated as described, of ∼1 µm width in the 50–90 keV range, are used for various experiments such as nf-HEDM, that provide 3D grain maps (with location, shape and orientation) of polycrystalline materials. This technique benefits from many millimetres long line foci, which the SRLs can deliver due to their large lateral aperture of up to 6 mm – a capability not offered by etched Si CRLs due to submillimetre etch depths.

## 5. Short focal length – Al lenses   

**Figure 5:**
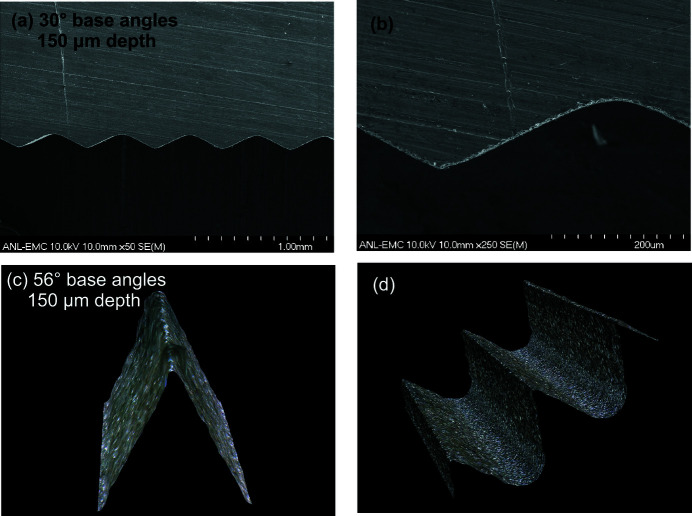
(*a*, *b*) SEM and (*c*, *d*) optical microscope images of Al saw-tooth lenses made by wire-EDM.

SRLs made from Al are also used at 1-ID, from having been explored as an alternative to the Si devices while a repeatable fabrication source and process for the latter was developed. The Al devices (Fig. 5 ▸) are made by electric discharge machining (EDM) using wire diameters in the 20–100 µm range, with different machining trajectories, tooth angles and Al types. Although the wire-EDM process was not expected to produce tooth profile tips and valleys having the same sharpness as the anisotropically etched Si devices, they were expected to be acceptable for the larger spot sizes of long focal distances and horizontal focusing at short focal distances. For purposes of comparative assessment among different lenses, the more stringent test configuration of vertical focusing at short focal lengths within the E station is employed. Furthermore, for efficiency and economy in examining performance under various SRL fabrication parameters, only an upright lens piece is tested. There is no need to test a combined upright/inverted SRL pair, as that entails the uninformative additional steps of optimizing the focus from a second piece (in addition to fabricating it) and steering the foci from the two pieces to coincide, an alignment procedure to be discussed later.

**Figure 6:**
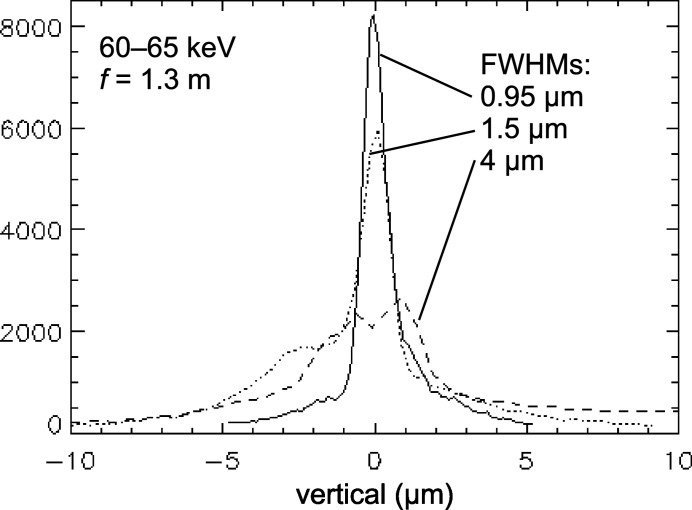
Vertical focus profiles for a short-focal-length configuration, with 60–65 keV X-rays focused by various Al lenses at 68–70 m in the E station.

Fig. 6 ▸ shows focus profiles from three Al SRLs tested (upright piece only) placed at 68.7 m in the E station, focusing vertically to the 70 m position (see Fig. 2 ▸), corresponding to *f* ≃ 1.3 m. A ∼ 60 keV X-ray beam of size 300 µm × 100 µm (H × V) was incident at grazing angle α ≃ 0.07° on each of the lenses, which were 16 cm long, with isosceles teeth of 150 µm height. The 4 µm (dashed line) and 1.5 µm wide (fine-dotted line) foci were obtained from Al SRLs with teeth having 520 µm period (30° base angles) cut with 100 µm wire. The narrower focus of these two was obtained by changing the EDM trajectory from a looping (circle-and-return at the tips) to a zigzag one. The third focus profile of 0.95 µm width (solid-line) was obtained by reducing the period to 202 µm (56° base angles) and the wire diameter to 50 µm. The measured flux density gain for this third lens (upright piece only) was 50, implying a potential gain of 100 if an upright/inverted pair were used. This is lower than the gain obtained with the single-crystal Si SRLs (previous section), likely due to the polycrystallinity and lower profile quality of the Al devices. Forming a more detailed understanding of how the EDM fabrication parameters influence final focusing performance is ongoing.

Figs. 5(*a*) and 5(*b*) ▸ show scanning electron microscope (SEM) images of the zigzag-cut, 30° base angle lens that focused to 1.5 µm. Figs. 5(*c*) and 5(*d*) ▸ show optical microscope images of the 56° base angle lens that focused to 0.95 µm. The radii on the tips and valleys are apparent, particularly on the 30° device cut with thicker wire. The rounded valleys can be excluded from being active in the focusing by reducing the incident beam size to prevent illumination of those regions. This was done here by using a 100 µm vertical beam, which is smaller than the 150 µm tooth height, resulting in only the straight-slope portions of the teeth being illuminated. On the other hand, not much can be done about the rounded tooth tips, which can be viewed primarily as missing-tip defects, resulting in some rays not receiving their full refractive deflections for proper focusing. For an upright lens piece, these mis-steered rays end up slightly below the main focal spot. This can be seen as shoulder features on the left sides of the beam profiles (Fig. 6 ▸) from the 30° base angle lenses. It should be pointed out that the different Al SRLs and the Si SRLs all perform comparably at long focal lengths, *e.g.* focusing from B to E stations.

## 6. Two-dimensional focusing   

To achieve point (2D) focusing with a full parabolic aperture, four SRL pieces are required – upright/inverted and left/right pairs for vertical and horizontal focusing, respectively. Despite the added motion control complexity, there are flexibility advantages to systems offering independent control of the two focusing directions. Sometimes an experimental technique makes use of a line focus (*e.g.* nf-HEDM), or angular collimation of the beam is needed in one plane only (*e.g.* vertically, at the 32 m location just before the high-resolution monochromator; see Fig. 2 ▸). Even when a 2D focus is called for, this might be done best from two separate locations for the vertical and horizontal focusing, to free oneself from the source size aspect ratio. This is done often at 1-ID, where horizontal focusing is done from the SRLs at 68–69 m and the vertical focusing is done from the SRLs at 32 m or 38 m, to deliver a less eccentric (*i.e.* more circular) beam. Another case for different demagnifications is to compensate for different effective vertical and horizontal source points that might occur either due to the storage ring lattice or upstream X-ray optics, such as intentionally prepared secondary sources.

**Figure 7:**
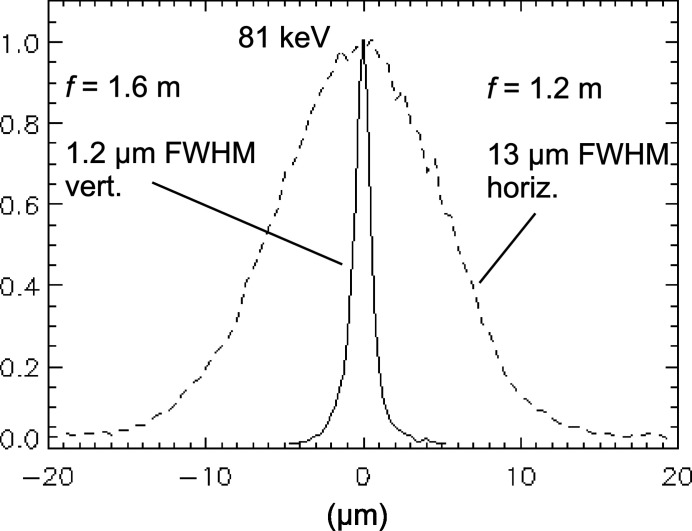
Vertical and horizontal profiles of an 81 keV point focus using a combination of Si and Al lenses at 68–70 m on four motion stage-stacks in the E station pictured in Fig. 9 ▸.

Fig. 7 ▸ shows the profiles of a 2D focus at 70 m in the E station with 81 keV X-rays, using a vertically focusing Si SRL pair at 68.4 m (*f* ≃ 1.6 m) and a horizontally focusing Al SRL pair at 68.8 m (*f* ≃ 1.2 m). The focal spot size was 13 µm × 1.2 µm (H × V). In this configuration, the approximately 100-fold flux density gain from vertical focusing is further augmented by a factor of six to ten from the horizontal focusing, depending on the quality of the horizontal SRLs. The horizontal lenses used here were a pair of the lowest quality Al lens type, which gave the widest focus of 4 µm in the vertical test (Fig. 6 ▸). The expected horizontal focus size is 11 µm.

## 7. Control, alignment and diagnostics   

**Figure 8:**
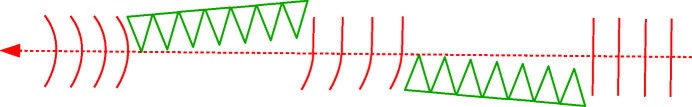
Illustration of wavefronts passing through a longitudinally offset saw-tooth lens pair, operating at slightly different focal lengths to establish convergence to a common focus.

**Figure 9:**
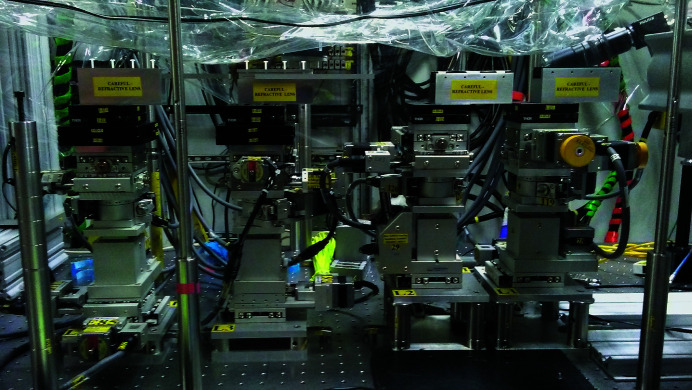
Four motion stage-stacks for 2D focusing with two saw-tooth lens pairs in the E station. The beam propagates from right to left, encountering (in order) the inverted, upright, out-facing and in-facing lenses, positioned at distances 1.7 m, 1.5 m, 1.3 m and 1.1 m, respectively, from the focal spot location at 70 m from the source.

Although Fig. 1 ▸ shows the pieces of an SRL pair at the same location along the beam, directly facing each other, such an arrangement is not essential. One can also operate with the lens pieces offset longitudinally along the beam as depicted in Fig 8 ▸. All the SRL set-ups at 1-ID use this offset scheme, as it facilitates the mounting and control of the lenses by having independent, separated stage-stacks conveniently under them. So the 2D focusing set-up in the E station is implemented by four sets of stages separated by 200 mm, positioned from the source (focus) at 68.3 m (1.7 m), 68.5 m (1.5 m), 68.7 m (1.3 m) and 68.9 m (1.1 m) distances for the inverted, upright, right and left SRLs, respectively (Fig. 9 ▸). As a result, the four elements operate at slightly different focal distances, and hence grazing angles. Kinematic mounts holding the lenses enable easy interchanging of relative placements of the vertical and horizontal focusing lenses to control focal spot properties, and even use other types of focusing optics such as CRLs and kinoforms. Since focal length can be continuously tuned by adjusting the grazing angle, translation along the beam is not needed. So for SRLs, in principle, each stage-stack needs to provide five degrees of freedom. However, the sixth degree of freedom is desirable for having the capability to employ CRLs or kinoforms.

The longitudinally offset placement of an SRL pair does not affect the coherent superposition of the two elements in the case of a coherent (*e.g.* point) source. This is clear from Fig. 8 ▸, which sketches the wavefronts as they propagate through the system, with the optical path length being the same for the two pieces. The SRL pair has a smaller focusing diffraction limit than a single piece, due to its doubled aperture. This is routinely observed in practice at 1-ID, with a pair giving a slightly smaller focus than either single element alone, due to the reduction in the diffraction limit contribution to the focus size. Consideration of these coherence and diffraction limit aspects of SRLs enters into their application to Bragg CDI, specifically regarding the size limit on a specimen grain within the focus to receive fully coherent illumination.

**Figure 10:**
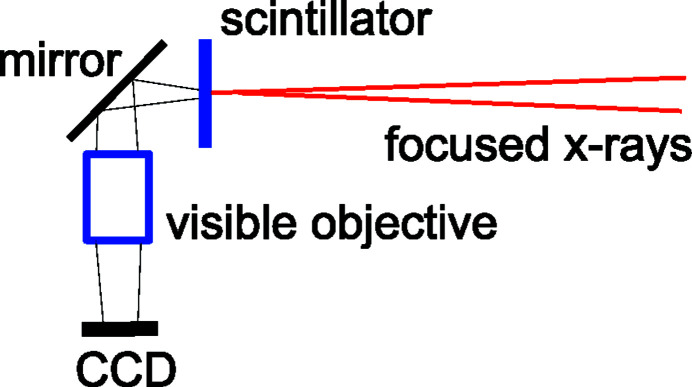
High-spatial-resolution beam imaging camera for fast focusing alignment.

**Figure 11:**
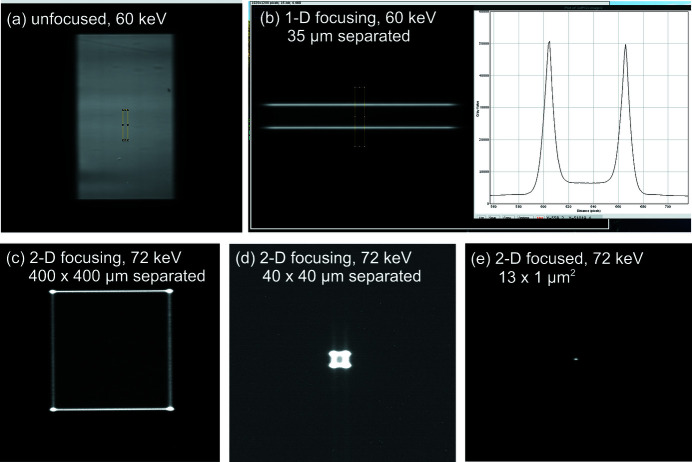
Images at the focal plane taken with the imaging camera (Fig. 10 ▸) during the procedure of (*a*, *b*) 1D vertical focusing with a pair of lenses and of (*c*–*e*) 2D focusing with two pairs of lenses.

Although the short-focal-length profiles shown here were obtained by fluorescence scans (Figs. 4 ▸, 6 ▸ and 7 ▸), fast optimization of the focusing for routine beamline operations for user experiments is done using a real-time beam imaging camera placed at the focal plane (Fig. 10 ▸). These cameras, developed for micro-tomography at high energies, consist of a thin Ce-doped LuAG scintillator, whose conversion to optical photons is reflected and imaged onto a CCD-array through a magnifying objective. With CCD or CMOS sensors having 5–8 µm size pixels (*e.g.* QImaging or Point Gray) and 5–10× objectives, X-ray imaging with ∼1 µm pixels is achieved, leading to a final spatial resolution of a few pixels, including blooming effects from a focused beam. It is important to note that, even though this resolution is insufficient to measure the detailed focal spot profiles of ∼1 µm beams, it is adequate to optimize the SRL focusing. One does this by maximizing the detected peak intensity in the line focus. Fig. 11(*a*) ▸ displays a camera image of an unfocused 300 µm × 500 µm (H × V) beam at the 70 m location in the E station. The image in Fig. 11(*b*) ▸ was taken after raising and lowering, respectively, the upright and inverted SRLs (at 68.4 m) into the beam at approximately the correct grazing angles for *f* ≃ 1.6 m focusing, showing the line foci from the two lens pieces and a vertical line-out profile. The grazing angle orientations of the lenses are then adjusted to maximize the peaks in the line-out profiles, thereby matching the individual focal distances to the camera’s scintillator position. Having the SRLs mounted so that the on-axis end-tooth is on the grazing-incidence rotation axis is desirable to minimize steering of the focus when changing this angle. Finally, one steers the two line foci in Fig. 11(*b*) ▸ into coincidence by vertically adjusting the SRL pair, achieving a single line focus at the center of the unfocused beam. Having tilt adjustments on the SRLs, *i.e.* rotations about the beam axis, is important to obtain exact parallelism between the line foci of an SRL pair, for the best merged focus. For 2D focusing, the first step is to optimize the horizontal focusing, in a manner similar to the procedure just described. One then separates the two SRLs to go back to the unfocused beam and optimizes the vertical focusing. Finally, the horizontal focusing is reintroduced by restoring the prior established positions. This procedure has the property of not disturbing the narrower vertical focus once it is achieved. Delivering 2D focusing takes about 30 min from an unaligned state at any energy, after which alternating between focused and unfocused beams takes a few seconds. Figs. 11(*c*) and 11(*d*) ▸ show images taken with the four SRLs spread out at different separations from the point-focused condition [Fig. 11(*e*) ▸].

**Figure 12:**
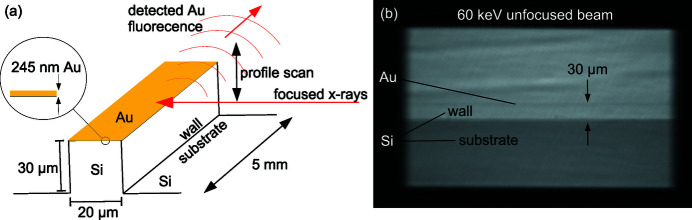
(*a*) Focal spot fluorescence profiler composed of a thin Au layer residing on a Si wall. (*b*) Its transmission in an unfocused beam, viewed by the imaging camera (Fig. 10 ▸) positioned 240 mm after it.

The 245 nm-tall Au wire, whose fluorescence was used for the high-quality focal profile scans, is actually a microfabricated bar of rectangular cross section 0.245 µm × 20 µm and 5 mm length, supported on a Si wall of dimensions 30 µm × 20 µm × 5000 µm, protruding out of a 5 mm × 5 mm Si substrate, as illustrated in Fig. 12(*a*) ▸. This structure was fabricated using standard photolithography and Si micromachining techniques. Steps involved coating and subsequent removal of protective patterned photoresist layers, sputter-deposition of Au, Si etching and finally Si dicing. A critically important advantage of this profiler is that the raw scan directly shows the focal profile. This is in contrast to knife-edge type profilers often employed by researchers, whose fluorescence scans have to be differentiated, giving lower quality profiles, mainly due to derivative artifacts arising from photon-counting statistics, which also change as the knife-edge is scanned through the beam. The high-quality focal diagnostics from the Au bar used here, revealing subtle features like asymmetries, shoulders and tails, have been invaluable in ongoing efforts to incrementally push the SRL focusing well into the submicrometre regime. There is also no scattering from the Si substrate, which the focused beam does not illuminate, since the Au is suspended away from it on the thin Si wall. When measuring the Au fluorescence in an unfocused beam, for flux density gain determination, the beam size is apertured to avoid hitting the substrate.

The imaging camera used for fast focusing alignment is also used to align the Au profiler. Fig. 12(*b*) ▸ shows the image of the unfocused beam transmitted through the profiler placed at the intended focal plane and the camera placed a few hundred millimetres beyond. A faint line formed by the thin Au bar, enhanced through phase contrast, is visible stretched horizontally across the image, 30 µm above the substrate. For optimal fluorescence scans, one takes care to align the bar’s 20 µm thickness dimension to be parallel to the beam by making the phase contrast artifact as narrow as possible. Using this imaging configuration, one can also park the Au profiler at the beam center, where the focal spot is to be directed by SRL alignment, in preparation for the scanning diagnostics.

## 8. Final remarks and nanofocusing prospects   

This article is aimed at communicating the APS 1-ID beamline’s experience and techniques in implementing high-energy X-ray focusing with SRLs, which perform very well but are not in widespread use. Their salient feature is having an effectively parabolic profile whose curvature is continuously tunable for energy or focal length. Although this is particularly valuable for tunable energy beamlines, fixed energy beamlines can also benefit from variability in the exact positioning of the focus, *e.g.* for instruments at different locations. Additionally, SRLs operate in-line, with no on-axis attenuation, and have large spatial acceptances in the direction perpendicular to focusing (for long line foci). Materials like Si and Al are well suited for SRLs operating at 50–100 keV X-ray energies due to the balance between refractive strength and attenuation.

Focusing of high-energy X-rays to small beam sizes is not as developed as for the case of lower energies, where nano­focusing is becoming routine. Focusing down to 1 µm at high energies is still non-trivial. As the energy increases from conventional hard X-rays (7–30 keV) to high-energy X-rays (50–100 keV), optics for efficient control of the radiation (*e.g.* monochromatization, analyzers, focusing) become more challenging. In focusing, shorter wavelengths lead to the requirement of increasingly finer features in fabricated structures, typically in the direction transverse to beam propagation. At the same time, the weakening of the interaction with matter, *i.e.* the refractive strength decreasing with the square of the wavelength, lengthens longitudinal dimensions. So depending on the device type, this leads to encountering problematic issues like smaller grazing angles, longer devices or longitudinally thicker structures, which in conjunction with finer lateral features present more difficult longitudinal-to-transverse fabrication aspect ratios.

Focusing down to ∼1 µm FWHM (vertically) has been shown here with SRLs. However, significant progress towards submicrometre focusing has been under way and will be reported elsewhere. Simulations show that the basic SRL concept, based on a linear, periodic, triangular structure, should ideally be capable of delivering vertically focused beams down to ∼650 nm in the 1-ID-E station at 70 m in the *f* = 1.3 m configuration. Focusing to just under 700 nm has been achieved with both Si and Al lenses. The Si lens improvements entailed care in wafer choice, processing and final mounting. The Al lens improvements were due to additional EDM refinements. Shortening the focal distance would further reduce the spot sizes, provided that it is accompanied by the mitigation of aberrations intrinsic to SRLs. Like all refractive optics, SRL focusing has contributions from source size, diffraction limit and chromatic aberration. However, they also possess two unique geometrical aberrations, each on the level of a few hundred nanometres. The contribution to the geometrical optics point spread function from the refractive discreteness of the teeth has already been mentioned earlier. The other contribution is a length aberration arising from the SRL not being a zero-length device but one whose refractive ray deflections from the teeth are distributed longitudinally over a distance that might not be negligible compared with the focal distance. The refractive discreteness aberration could be addressed by an adaptive optic that corrects for the effect (Seiboth *et al.*, 2017 ▸). The length aberration can be addressed by adjusting the total refractive deflection as a function of the incoming ray’s off-axis position (*y* in Fig. 1 ▸), *e.g.* by bending the device or varying the saw-tooth period over the length.
